# Barriers to Chagas Disease Screening among Primary Care Providers in the United States

**DOI:** 10.4269/ajtmh.25-0559

**Published:** 2026-03-10

**Authors:** Malwina N. Carrion, Rachel M. Kienle, Daniel Bourque, Mari-Lynn Drainoni, Natasha S. Hochberg, Madolyn Dauphinais, Taylor Mello, Paula E. Stigler Granados, Davidson H. Hamer

**Affiliations:** ^1^ Department of Global Health, Boston University School of Public Health, Boston, Massachusetts, USA;; ^2^ Boston University Chobanian & Avedisian School of Medicine, Boston, Massachusetts, USA;; ^3^ Section of Infectious Diseases, Department of Medicine, Boston University Chobanian & Avedisian School of Medicine, Boston, Massachusetts, USA;; ^4^ Department of Health Law Policy & Management, Boston University School of Public Health, Boston, Massachusetts, USA;; ^5^ Evans Center for Implementation and Improvement Sciences, Department of Medicine, Boston University Chobanian & Avedisian School of Medicine, Boston, Massachusetts, USA;; ^6^ UMass Chan Medical School, Worcester, Massachusetts, USA;; ^7^ Department of Environmental Health, School of Public Health, San Diego State University, San Diego, California, USA;; ^8^ Boston University Center on Emerging Infectious Diseases, Boston, Massachusetts, USA

## Abstract

Chagas disease is a neglected tropical disease endemic to Latin America with approximately 10.5 million people infected worldwide. About 288,000 people live with Chagas disease in the United States, where it is officially not considered endemic and where screening is infrequent. This study aimed to identify barriers to routine Chagas disease screening among health care providers (HCPs) in U.S. primary care settings. This qualitative study consisted of semistructured individual and group interviews guided by the Theoretical Framework of Acceptability and the Health Belief Model frameworks. Between April 2021 and January 2022, 18 individual interviews and 10 group interviews were conducted virtually with physicians, nurse practitioners, and clinical support staff. Deductive thematic analysis was performed using NVivo software v. 11.0. Three major themes emerged. 1) Successful Chagas disease screening requires integration into clinical workflows and strong support from guidelines, 2) limited knowledge Chagas disease affects the likelihood of HCPs to screen, and 3) patients face many obstacles that need to be mitigated (e.g., lack of insurance, language barrier, and testing/treatment costs) before implementing a screening program. Barriers to implementing routine screening for Chagas disease in U.S. primary care settings exist but are modifiable. Screening for Chagas disease could be improved by raising awareness and interest among HCPs and affected communities. A key finding was that embedding screening into workflows through the use of electronic medical records is needed. These findings could lead to appropriate measures to address this gap in care, including enhanced screening and treatment of Chagas disease.

## INTRODUCTION

Chagas disease, a vector-borne infection caused by the protozoan parasite *Trypanosoma cruzi*, was first described by the Brazilian physician Carlos Chagas in 1909 after he was introduced to the vector, the triatomine bug.^1^^,^^2^ Chagas disease is endemic to Mexico and Central and South America (excluding the Caribbean islands) and is considered a neglected tropical disease.^3^^,^^4^ An estimated 100 million people are at risk for Chagas disease, and approximately 10.5 million people worldwide are currently infected.^3^^,^^5^^,^^6^ Chagas disease is spread primarily through the feces of an infected triatomine bug, although it can also be transmitted congenitally (from mother to child), through consumption of contaminated food and drink, and through blood transfusions and organ transplants from individuals with Chagas disease. Chagas disease has two phases of infection beginning with an initial acute phase that presents as a self-limiting febrile disease for 4–8 weeks. Treatment can then limit the acute phase from transforming into the chronic phase. However, left untreated, Chagas disease will develop into a determinate form of chronic disease in 30–40% of patients. Chronic Chagas disease is characterized by cardiac, digestive, or cardiodigestive issues, which can lead to sudden death.^3^^,^^7^

Although Chagas disease is not considered endemic to the United States by the CDC and the WHO, a recent perspective suggests that the disease may be endemic.^8^ Official estimates put the number of cases at about 288,000 people.^9^^,^^10^ This is likely to be an underestimate because of a lack of awareness about the disease, resulting in few health care providers (HCPs) screening their patients.^11^ Even among infectious disease (ID) specialists, knowledge about screening is relatively low.^11^^,^^12^ Moreover, many at-risk patients are often of lower socioeconomic status, immigrants, and/or migrants, and therefore, they may face barriers to accessing care in the first place, much less specialists, such as ID. The HCPs who are most likely to see high-risk patients are usually those working in general internal medicine (GIM), family medicine (FM), pediatrics, and obstetrics and gynecology (OB/GYN). Two separate studies found that in the United States, OB/GYN HCPs had the lowest level of Chagas disease knowledge among HCPs surveyed.^11^^,^^13^ In one study, 47% of OB/GYN specialists had never heard of Chagas disease, and 60% never considered the risk of Chagas disease in their patients.^11^ Cardiologists and primary care physicians (PCPs) broadly working in different specialties also had low levels of knowledge.^11^^,^^13^ Each year from 2011 to 2018, when treatment was only available through the CDC, they released only enough benznidazole (one of two Food and Drug Administration [FDA]-approved medications for Chagas disease) to treat ∼55 people.^14^ These findings suggest that only <0.3% of identified infected people in the United States received treatment, underscoring a critical lack of awareness of Chagas disease in HCPs and the resulting impact felt by their patients through undertreatment.^15^

Currently, no official guidelines exist from the CDC or professional organizations, including the Infectious Diseases Society of America, the American College of Obstetricians and Gynecologists (ACOG), the American Academy of Family Physicians (AAFP), and the American Medical Association, for routine Chagas screening, even among high-risk populations in the United States. One reason may be a lack of data to show that screening for Chagas disease in the United States is needed. In 2022, expert opinion screening recommendations were published as HCPs and public health professionals in the United States recognized the need for guidance on this growing issue.^16^ By contrast, blood donor screening became nearly universal because of FDA recommendations, starting in January of 2007.^17^

Despite low screening rates, evidence confirms the presence of Chagas disease in the United States. The Strong Hearts Program at East Boston Neighborhood Health Center (EBNHC) in Massachusetts launched in 2018 and aims to integrate screening for Chagas disease into regular primary care visits for adults, children, and pregnant women (during prenatal care) from endemic countries. As of the end of 2023, the program had screened over 10,000 individuals and identified a prevalence of approximately 1% or 100 previously undiagnosed cases.^18^ Although a significant portion of EBNHC’s patient population is considered high risk because of their or their mother’s country of origin, the observed 1% prevalence still exceeds rates observed in many endemic countries.^9^ Only a handful of countries have similar or higher average prevalence rates, such as Bolivia (6.1%), Colombia (1.0%), Ecuador (1.4%), El Salvador (1.3%), Guatemala (1.2%), and Paraguay (2.1%).^9^ This underscores the need for targeted screening interventions in high-risk U.S. communities. To our knowledge, the Strong Hearts Program is the first and only primary care-based routine Chagas disease screening program in the United States.

Given the limited knowledge of Chagas disease among HCPs and the lack of screening, further exploration of the barriers of screening practices is warranted. The purpose of this qualitative study was to better understand what HCPs see as the challenges to routine Chagas disease screening in the United States. We aimed to identify specific practical barriers to implementation and how these vary by HCP type, clinical setting, and geographical location in the United States. In addition, because Chagas disease disproportionately affects those of lower socioeconomic status and immigrant and migrant populations, we wanted to see whether there were any perceived barriers associated with these. The results of this study were used to develop a handbook on enacting routine Chagas disease screening that attempts to preemptively address the limitations identified.

## MATERIALS AND METHODS

### Study design and interview guides.

The individual and group interviews were used to answer the research question: “What are the barriers and challenges to setting up a routine Chagas disease screening program in a primary care setting in the United States?” Both semistructured interview guides were guided by the Theoretical Framework of Availability (TFA) and the Health Belief Model (HBM) framework. The TFA states that understanding the acceptability of an intervention is critical to ensuring successful implementation.^19^ The HBM addresses perceptions of susceptibility to disease, severity of disease, benefits (of testing and diagnosing), barriers, self-efficacy, and then, cues that can propel action.^20^ The interview guides can be found in Supplemental Appendix A (individual interview guide) and Supplemental Appendix B (group interview guide), including mapping of the questions to the TFA and HBM. During the individual interviews, HCPs were asked about their experiences and views on the Strong Hearts Program, including its strengths and weaknesses, ease of integrating the program into practice, and lessons learned that they thought would be useful for others replicating the program.

The group interviews consisted of one to four participants and on average, lasted 57 minutes, with a range of 30–79 minutes. Smaller than typical group sizes were planned based on the research team’s prior experience with conducting group interviews over Zoom. Groups with more than six participants were usually found to feel impersonal and led to less participation because of increased distractions and making participants feel less comfortable sharing their opinions.

### Participant recruitment.

We recruited two groups of participants for the different interview formats: 1) in-depth individual interviews with personnel familiar with the Strong Hearts Program and/or employed at EBNHC and 2) group interviews with personnel in the GIM, FM, pediatrics, and OB/GYN departments in parts of the United States with a high likelihood of encountering at-risk individuals. This includes individuals who work with populations that have a high rate of immigration from countries where Chagas disease is endemic.

Inclusion criteria for the individual interviews were as follows: any personnel at EBNHC or Boston Medical Center (BMC) who knew of the Strong Hearts Program and were 18 years of age or older. Individuals who declined recording of the interview or who could not be scheduled after three attempts were excluded. In addition to EBNHC providers, interviews were carried out with two physicians who designed and were integral to the start of the program (but are not physicians at EBNHC) as well as two ID specialists who saw patients referred from EBNHC to BMC, a safety-net and research hospital located in Boston, MA.

Inclusion criteria for the group interviews were as follows: any HCP or clinic/hospital administrator who worked in a community with patients at risk for having Chagas disease, was 18 years of age or older, and resided in the United States. Individuals who declined recording of the interview or who could not be scheduled after three attempts were excluded. Group interviews were organized by profession (medical doctors [MDs], physician assistants [PAs], nurse practitioners [NPs], and registered nurses) to encourage participants to share their experiences freely.

For the individual interviews, considering the work of Guest et al.^21^ on saturation as well as practical considerations for the size of both the Strong Hearts Program and EBNHC, 29 people were contacted to participate and 19 were interviewed using a convenience sampling approach. Individual interview participants were 12 MDs, 4 NPs, 1 pharmacist, 1 individual with a master of business administration degree, and 1 individual with a master of social work (MSW) degree. Participants were from the fields of ID, laboratory management, GIM, FM, and OB/GYN. Interviews lasted a mean of 45 minutes, with a range of 26–120 minutes. The MSW interview was lost before transcription and therefore, excluded from the results. There were 18 interviews included in the final analysis. By the 18th interview, little to no new information was being gained; therefore, no additional participants were recruited (the 19th participant was already scheduled before the 18th interview taking place).

For the group interviews, 43 HCPs were contacted directly by the project team to participate, and an estimated 75 more were reached by recruitment e-mails shared by participants with their colleagues. In addition, the project team’s X (Twitter) account (@BostonChagas) posted multiple tweets between July 2021 and November 2021 to recruit additional participants. Although some HCPs did reach out in response to the tweets, none met the inclusion criteria.

### Data collection.

Interviews took place between April 2021 and January 2022. All interviews were recorded through the Zoom platform and then transcribed by the research team. Individual and group interviews included an interviewer or facilitator and a notetaker. Participant names were recorded in a secure document along with an identification number. Only the participant identification number was recorded on the actual notes, files, and transcribed documents from any interviews. To protect the identity of group interview participants, individuals were asked not to share their names. Additionally, study staff indicated each participant’s identification number as their display “name” on Zoom before any other participants meeting them. Participants were offered a one-time $50 physical gift card as compensation for participation in either the individual interview or the group interview; eight participants declined the gift card.

Although 10 group interviews were planned and held, the number of participants in some group interviews was lower than expected despite attempts to recruit using multiple avenues (social media, direct connection, flyers and e-mails sent through department and program e-mail lists, etc.). Thirty-two people had initially agreed to participate, but seven canceled at the last minute or failed to show up without notice. This resulted in two “group” interviews consisting of only one person. As both the inclusion criteria and interview guide differed between the group and individual interviews, these single-person interviews were kept in the group interview category. No participants were eligible to take part in both the individual and group interviews, so all participants in this study are unique.

### Data analysis.

Deductive analysis was used for both individual and group interviews, and NVivo software v.11.0 (QSR International, Burlington, MA) was used for data management, coding, and analysis. The codebook was developed using the interview guides, which were based on the TFA and HBM, as well as from the researchers’ professional experience in the field while allowing for any emerging themes to be added.^19^^,^^20^ All interviews, both individual and group, were coded using the same codebook, which was imported into Nvivo, and they were coded under four parent nodes with multiple child codes: Workplace factors, external factors, personal factors, and patient factors. The parent nodes were named based on the themes that best encompassed the child codes. Two additional child codes were added as they emerged during the interviews (electronic medical record [EMR] under workplace factors and stigma under patient factors). Transcripts were reviewed again with the addition of these codes to ensure that these new codes were accurately captured.

One individual interview transcript and two group interview transcripts were each coded by three team members independently (the principal investigator and two research assistants). Once completed, the principal investigator and the two research assistants met to review discrepancies, discuss necessary changes to the codebook, and identify any emerging codes from the transcripts. Because of the high level of agreement between the three initial coders, once the revisions to the codebook were complete, the principal investigator coded and analyzed the remaining transcripts. One code, “opportunity cost,” ended up not being used during the coding process because the concept was never mentioned by any participants. The two researchers who were involved in the Strong Hearts Program were not involved in any data analysis or interpretation of results.

A frequency analysis was conducted to identify which of the codes were mentioned most often by participants during the individual and group interviews as well as the prevalence of each code (Table 1). These codes were then used to deduce what barriers and challenges participants saw to setting up a routine Chagas diseases screening program in a primary care setting in the United States.

**Table 1:** Frequency analysis of codes stratified into external, patient, personal, and workplace factors

Code	No. of Coding References	No. of Items Coded	Prevalence
External factors/guidelines	8	5	11/43 (26%)
External factors/medication	1	1	1/43 (2%)
External factors/professional societies	15	8	13/43 (30%)
Patient factors/ability to access health care	20	8	14/43 (33%)
Patient factors/cascade of care	10	8	10/43 (23%)
Patient factors/Chagas awareness	15	7	9/43 (21%)
Patient factors/insurance	26	11	20/43 (47%)
Patient factors/language barrier	12	6	12/43 (28%)
Patient factors/literacy	13	5	10/43 (23%)
Patient factors/stigma	8	5	7/43 (16%)
Personal factors/affective attitude	26	12	16/43 (37%)
Personal factors/Chagas knowledge	33	12	19/43 (44%)
Personal factors/coherence	22	10	15/43 (35%)
Personal factors/opportunity cost	0	0	0/43 (0%)
Personal factors/perceived effectiveness	16	9	12/43 (28%)
Personal factors/self-efficacy	13	6	9/43 (21%)
Workplace factors/additional workload	31	8	11/43 (26%)
Workplace factors/appointment length	2	2	2/43 (5%)
Workplace factors/communication in the workplace	19	8	10/43 (23%)
Workplace factors/communication with the CDC	7	4	4/43 (9%)
Workplace factors/EMR	18	5	5/43 (12%)
Workplace factors/follow-up care	1	1	1/43 (2%)
Workplace factors/point person	35	8	9/43 (21%)
Workplace factors/training	18	7	8/43 (19%)
Workplace factors/workflow	63	10	17/43 (40%)

EMR = electronic medical record. The number of coding references indicates the number of times that the code appears across all transcripts. The number of items coded indicates the number of individual documents in which the code appears. Prevalence indicates the percentage of participants whose response was coded in this category at least once during an interview.

## RESULTS

In total, 25 PCPs from four different states (Massachusetts, California, Florida, and New York) participated; their professions, specialties, and locations are shown in Table 2. Overall, results showed that many PCPs in the United States have similar views on barriers to routine Chagas screening in the United States. When there were discordant views, this was usually related to the PCP’s workplace (e.g., hospital or clinic). For example, many PCPs reported that insurance was a barrier for their patients, but those who work in integrated managed care consortia (like Kaiser Permanente) did not encounter this with their patients because all patients in the system have some type of insurance.

**Table 2:** Group interview participants

Group	Participant	Title	Specialty	State	Participant	Title	Specialty	State
1	1	MD	GIM	MA	3	MD	FM	FL
	2	MD	FM	FL	–	–	–	–
2	4	MD	PEDS ID	MA	6	MD	GIM	CA
	5	MD	FM	MA	–	–	–	–
3	7	MD	PEDS	MA	–	–	–	–
4	8	MD	FM	CA	10	MD	FM	MA
	9	MD	FM	CA	11	MD	FM	CA
5	12	MD	ID	NY	14	MD	UC	CA
	13	MD	FM	MA	–	–	–	–
6	15	RN	GIM	MA	16	RN	OB/GYN	MA
7	17	PA	EM	CA	18	PA	EM	CA
8	19	MD	PEDS	MA	–	–	–	–
9	20	MD	OB/GYN	MA	22	MD	ID	MA
	21	MD	GIM	CA	23	MD	GIM	MA
10	24	NP	GIM	NY	24	NP	GIM	NY

CA = California; EM = emergency medicine; FL = Florida; FM = family medicine; GIM = general internal medicine; ID = infectious disease; MA = Massachusetts; MD = medical doctor; NP = nurse practitioner; NY = New York; OB/GYN = obstetrics/gynecology; PA = physician assistant; PEDS = pediatrics; PEDS ID = pediatric infectious disease; RN = registered nurse; UC = urgent care.

Three major themes emerged from the interviews. 1) Successful Chagas disease screening requires integration into clinical workflows and strong support from guidelines. 2) A lack of knowledge about Chagas disease affects the likelihood of HCPs screening. 3) Patients face many obstacles that need to be mitigated (e.g., lack of insurance) before implementing a screening program. These themes were consistent across both the individual and group interviews. Each theme is described below along with quotes from the participants. GD refers to group interview, and it is followed by the group number and then, the participant number (GD1-2 is group interview 1, participant 2 in that group). Overall, data from 43 unique participants were included in the results.

### Theme 1: For the implementation of a Chagas screening program to be successful, it needs to be integrated into workflow and supported by guidelines.

The importance of integration of screening for Chagas disease into regular workflow was a strong theme throughout all individual and group interviews. Many HCPs stated that they would find it hard to add Chagas disease screening to their practice if it was not in an order set or included as part of their screening for other diseases. Participants also expressed the need for support tools, such as EMR “smart phrases” or prompts, to assist them in remembering who to screen, how to explain Chagas disease to patients, and what to do if a patient screened positive.

*“[We need], not just teaching, but just saying “Okay here’s … here’s an order set with … you know, what you need to know about kind of a range of similar topics.” Also associated with a smart phrase, so that when … when the primary care doctor gets their results, [they] know what to do. So, if it’s positive, do I consult ID for this thing? Or you know, for that slew of tests you’re asking me to do, with … with next step.”* —GD9-3

*“I have a patient panel, about 1,900 patients and about half of them are Spanish speaking and I live in an area where there’s a large Central American population. So, probably half of my visits per day are potentially people who could be in a group [i.e., have Chagas disease]. So, that workflow of how it’s done along with other tests if it’s all ages or not, what the follow up in the workflow would end up being, and how the lab would coordinate with CDC would sort of be imperative for … for me to be able to roll it out.”* —GD9-2

HCPs noted that involving staff, such as nurses or medical assistants, in screening would help ensure that it was routinely done. Health care providers at EBNHC also added that having a patient navigator had made a major difference in ensuring that their patients remembered their appointments and felt supported by the clinic after diagnosis.

*“I would add that whenever we’ve implemented a program like this, having my staff like, the back-office staff or my nurse, be one of the screeners where they’re asking those certain questions. She could pend the screening program and that just kind of triggers me to ask about that. So having the staff help out would be important.”* —GD4-2

*“Like basically, the nurse or whatever says “Oh, this person is from an endemic country that you know Epic says, you know this person would be due—has never had the Chagas thing” and then it says like “Okay, order to screen it.” I can’t … I don’t imagine that in my practice I would say “Oh, this person is from Latin America, I’m going to order the Chagas screening tests.” And that may just be due to my ignorance, but I think it would have to be probably beyond just basic education for me to start ordering screening tests on all my patients, because that’s far from what I do currently.”* —GD4-1

Finally, many participants observed that the influence of official guidelines (whether from the CDC or a professional society) would have a large impact on their likelihood of incorporating screening for Chagas disease into their workflow. According to one physician,

*“Certainly [we would follow it], like if it were to become something like the [U.S. Preventative Services Task Force]. “Oh, if you are, you know, if you are from this area, you know, you should get screened for it.” And certainly, right? Because that’s what we … that’s what we follow and that’s also what we teach our students.”* —GD1-1

Some participants noted that official guidelines would be more influential even than their own impressions of the disease or motivation to screen. As one physician put it,

*“I mean, it wouldn’t even matter if [screening for Chagas disease] were important to me or not. It would … it would matter [if ACOG issues guidelines].”* —GD6-2

Overall, workflow integration, including having professional guidelines in place to support screening, was the major theme that emerged from the interviews and was universal among all participants.

### Theme 2: A lack of knowledge about Chagas disease is a concern for HCPs.

Health care providers in both the individual and group interviews felt that their lack of knowledge about Chagas disease might be a barrier to their participation in a screening program. Several participants mentioned that they had received very little training about Chagas disease and that they would not feel confident to speak to a patient or to colleagues about it without additional support. Some HCPs also acknowledged that they currently work with at-risk patients but do not screen because of their own lack of knowledge as well as a lack of emphasis on screening at their institution.

*“[Lack of knowledge about Chagas disease] would be a barrier for me. I don’t think … I don’t feel very well versed in Chagas disease.”* —GD6-1

*“I learned about Chagas disease in med school, but I haven’t really considered screening for it since I started residency, and I do sometimes work with patients who are immigrants from Latin American countries.”* —GD3-1

### Theme 3: Patients face many obstacles that will need to be addressed.

Most HCPs expressed worry about the different obstacles that their patients might face when screening for Chagas disease along with subsequent diagnosis and treatment. Concern around insurance was the most prominent topic, with patients often being uninsured or underinsured. Health care providers feared that the costs of initial screening, subsequent testing, and treatment may produce financial hardships for their patients without any clear or accessible solutions.

*“The Affordable Care Act obviously changed a lot with our second-generation migrant patients in terms of insurance. But, when you’re talking about our undocumented population you know, insurance isn’t even an issue. I mean they don’t have the opportunity to be insured period. They don’t have papers so.”* —GD10-1

*“[Routine screening for Chagas disease would be] a little hard where I work, I would say, just because we have a lot of immigrant population that’s uninsured, completely undocumented. So, like that first screening sample would come out of pocket so, like negotiating costs, with the other companies like the lab companies will be part of the equation for us, and obviously if the cost is manageable then it would make it easier. Because most of the times when I see patients who are undocumented, they usually say like “what is that gonna cost me?”* —GD5-2

*“So, apart from the preliminary tests being done, let’s just presume that gets done. The follow up testing, I think is where in our clinical setting it gets problematic. Where, if you need a chest X-ray, patients have to pay out of pocket for them. This is, once again, we’re dealing with a 100% uninsured patient population in my clinic and if you need an echo, it gets all the more expensive and it gets very difficult to navigate these studies. So, that serves as somewhat of a disincentive for our patients to follow through on these tests.”* —GD1-2

Besides insurance, there were broader concerns about accessing care, such as transportation and other logistics.

*“Well, you know, screening is great, but if you screen positive then there’s the “dot dot dot.” But the challenge then, to get this person down to New York City, where the one place that was a potential treatment venue. So, you know, that’s the other thing, I think. Whenever we talk about screening is, we have to think about next steps.”* —GD10-1

*“Yeah, it is hard, I think, in general, to have patients follow up with specialists. Logistically, sometimes the appointment schedulers aren’t able to get a hold of the patient. Sometimes they just try multiple times, and they just aren’t able to get a hold of the patient. If the appointment is scheduled sometimes the patient isn’t aware of it or they just don’t attend the appointment, so it is difficult, just in general to schedule appointments at specialists.”* —GD2-3

Finally, language barriers, literacy, and stigma were also commonly mentioned across both individual and group interviews.

*“In terms of Chagas, I think it’s a difficult situation, even once you have the diagnosis, because again language is often an issue and … and even interpreters cannot find the right words, even for the illness, Chagas, to connect with a patient and tell them what … what it is. So, this I find particularly challenging, and I almost feel like it needs its own … we need like a specialist that can you know, understand … like the interpreter has to understand what is Chagas and what it’s called in the location of the patient. I think it’s something that needs to be thought out in [the case of Chagas disease].”* —GD9-3

*“In other words, sometimes our immigrant patients want to kind of stay below the radar. And I think it might just take a little bit of … it might be slow in the beginning [for fear of stigma to subside] until it becomes more common and maybe people have heard about it from their neighbors or their relatives that you know, it’s nothing to feel scared about and no one’s going to be reporting you or anything like that. Then I think [screening for Chagas disease] might be more successful.”* —GD6-2

## DISCUSSION

This study identified several key challenges and barriers to integrating routine screening for Chagas disease into primary care from the HCP perspective. It also yielded information that can be used to inform initiatives to implement screening programs. The results revealed the importance of aspects such as guidelines and the role of insurance.

The most salient findings were the need to integrate screening into existing workflows as opposed to putting the burden on HCPs to remember to screen. For example, if the screening test was included on a panel of tests that the HCP was already ordering, particularly within an EMR, providers would be more likely to consider screening a patient for Chagas disease.^22^ Likewise, participants expressed the need for support from other staff and the helpfulness of having a point person or a patient navigator in ensuring sustainability of the program. A point person or a central person to coordinate screening results is helpful because that person removes some of the burden on the HCPs to follow-up with patients, deal with referrals, and maintain a high level of Chagas disease knowledge.^22^ This would both remove the requirement of a provider to identify high-risk patients and potentially remove the stigma in being asked to screen for the disease. A patient navigator can help those struggling with access to health care or being able to attend their appointments as many of the participants’ patients do. Although adding responsibilities to staff or hiring a patient navigator may incur additional costs, these roles seem to be an essential part for successful implementation of a screening program. If the burden is removed from the primary care provider, this could potentially be cost saving. Although resource availability and allocation remain health care cost concerns, the benefits to improved outcomes because of screening are likely to outweigh the costs. The addition of an EMR alert for screening for hepatitis C virus (HCV) and HIV helped to improve screening rates in a primary care practice within a large health care system.^23^ Helping to address health care equity for at-risk individuals can also be a motivating factor.

The influence of official guidelines and/or recommendations from professional societies was mentioned often, particularly among group interview participants. Many participants said that ultimately, what they screen for or which screenings are adapted by their workplace are driven by these guidelines and recommendations. This further solidifies the need for national guidelines, ideally from the CDC or the U.S. Preventative Services Task Force, as well as support for screening across multiple professional societies, such as the American Society of Tropical Medicine and Hygiene and the AAFP. Currently, a circularity exists in the United States whereby organizations may be reluctant to develop guidelines because of insufficient screening data to serve as an evidence base. However, as shown in this study, the lack of guidelines results in HCPs not screening their patients and generating the epidemiological data needed for an evidence base. Therefore, there needs to be a strong effort to encourage motivated HCPs to screen despite the lack of guidelines. Screening all patients can decrease stigma associated with a disease as well as allow for enhanced linkage to care as shown by EMR screening alerts for HCV and HIV.^23^ As screening generates the epidemiological data, this may prompt the development of official guidelines, which will, in turn, encourage more HCPs to screen. Advocacy by the patients, communities, and HCPs also might propel societies to write these guidelines if they hear the same message from many different affected parties. In addition, more cost–benefit analyses are needed to show both the cost savings that could be achieved through increased and earlier diagnoses as well as through using specific supports, such as patient navigators, to keep patients on treatment.

Although none of the participants in the group interviews currently conduct routine screening for Chagas disease, many recognized or came to recognize the importance of doing so for their patient population. However, the results clearly indicated that a lack of disease awareness among HCPs continues to be an immense barrier to screening. Many group interviewees acknowledged limited familiarity and experience with Chagas disease, including screening protocols and appropriate steps to take if a patient does screen positive. This highlights the ongoing need to raise awareness and provide education among HCPs across the United States. Additionally, medical schools should consider updating their curricula to reflect the needs of a changing population that may be increasingly at risk for Chagas disease. On a positive note, participants expressed interest and enthusiasm in learning more about Chagas disease and recognized it as a current gap in their training.

Finally, this study highlights that immigrant and migrant populations face numerous barriers accessing health care in the United States. Being underinsured or uninsured creates a substantial burden on both patients and the health care system. Although not unique to Chagas disease, it is especially relevant because high-risk populations tend to be people with undocumented status, people who may be experiencing poverty, and/or people who may be uninsured or underinsured. Insurance was brought up in interviews as an issue from both the provider side and the patient side. The HCPs may be reluctant to screen patients for a disease that could be costly to diagnose and treat. Simultaneously, HCPs feared that their patients may refuse screening or following up on a positive result because of the associated costs. Other important barriers mentioned included stigma, literacy, and language barriers. Interestingly, some HCPs noted that even having medical interpreters is often not enough to effectively communicate about Chagas disease with patients. This is because of the complexity of the disease and because much of the associated vocabulary has regional differences across Latin America.

This study has several major strengths and limitations. The strengths include a relatively large sample size, particularly given the limitations of online interviews during the coronavirus disease 2019 pandemic, and a mixed group of respondents encompassing both those currently screening for Chagas disease and those who could be the primary audience for future screening programs. There was also some geographical variation and a mix of specialties, HCP roles, and work settings, which allowed us to gain a broader understanding of screening challenges in different health care settings. Participants were engaged during the interviews and enthusiastic about participating and sharing their opinions, even when admitting a lack of knowledge. Finally, the use of the TFA and the HBM as the theoretical grounding for the study helped to produce clearer and more robust information.

Limitations include the fact that we were unable to recruit HCPs at EBNHC who did not participate in the Strong Hearts Program, despite repeated efforts. This may reflect the fact that nearly all HCPs at EBNHC do participate in the Strong Hearts Program, but it also highlights the need to understand why HCPs surrounded by colleagues who do screen for Chagas disease choose not to routinely screen. Although the sample size was considered a strength because it was (purposefully) so diverse across specialties, it means that it was not meaningful to analyze the data by specialty. Finally, although the intention was to recruit a broad range of participants, those who chose to participate are likely more interested in Chagas disease or screening practices. For example, there were no suitable recruitments from the efforts made on X (Twitter). This and therefore, a reliance on HCP networks may have led to selection bias, creating a population that could be less likely to perceive certain factors as barriers to screening because of their interest in overcoming them. This also limits the generalizability of the results to the broader HCP population.

## CONCLUSION

This study looked to identify challenges to implementing screening for Chagas disease in the primary care setting. Although the findings were in line with the limited literature, the frequency of some of the concerns and gaps being mentioned was greater than anticipated. For example, there were varied levels of concerns about screening costs, which highlighted the differences among health insurance coverage from state to state. The impact of a lack of national screening guidelines and the willingness by some providers to change their practice if guidelines were issued are surprisingly strong. Overall, this study showed that lack of awareness about Chagas disease is still a major obstacle both among HCPs and in affected communities. However, HCPs serving at-risk populations are interested and willing to consider implementing routine screening for Chagas disease into their practice. The sharing of resources, such as the handbook created by the Implementing Novel Strategies for Education and Chagas Testing team that was developed using the results of this study^24^ or EMR resources across screening sites, might help ease the burden for these HCPs. Nonetheless, greater efforts are needed to raise the importance of Chagas disease to the national stage, particularly as U.S. demographics change and the threat of autochthonous transmission increases. Future research should continue with both qualitative and quantitative studies to further explore awareness of Chagas disease and whether there are other barriers that can be addressed to increase screening and to validate this study’s findings. Large quantitative surveys could help with shining a light on the need for increased awareness and training of PCPs on Chagas disease, whereas qualitative studies, especially in other high-risk communities, could potentially reinforce some of the barriers identified in this study (and therefore, the need for systematic changes) and also identify new barriers specific to certain environments and populations to better tailor the implementation of screening activities.

## Supplemental Materials

10.4269/ajtmh.25-0559 Supplemental Materials
