# Supplementary material for: Barriers to Chagas Disease Screening among Primary Care Providers in the United States
Source: Am J Trop Med Hyg. 2026 Mar 10;114(5):857–64. doi: 10.4269/ajtmh.25-0559 (PMC13153594; doi:10.4269/ajtmh.25-0559)
Supplement: Supplemental Materials [file tpmd250559.SD1.pdf]

Strong Hearts Evaluation In-Depth Interview Guide

I want to thank you for taking the time to meet with me today. My name is ______________________________ and I would like to talk to you about your experiences with the Strong Hearts program at East Boston Neighborhood Health Center. Specifically, we are hoping to learn more about the strengths of the program and any barriers to implementing it that you have come across. We hope to use this information to help other health centers and hospitals roll out similar programs.

The interview should take less than an hour. I will be recording the session because I don’t want to miss any of your comments. I will be taking some notes during the session, as well.

All responses will be kept confidential. This means that your interview responses will only be shared with research team members and we will ensure that any information we include in our report does not identify you as the respondent. Remember, you don’t have to talk about anything you don’t want to discuss, and you may end the interview at any time.

Are there any questions about what I have just explained? Are you willing to participate in this interview?

Questions

1. What is your position at East Boston Neighborhood Health Center (EBNHC)/Boston Medical Center (BMC)?

2. How long have you been working there?

3. What has been your involvement in the Strong Hearts program?

4. Can you please explain to me your understanding of the aims of the program and how it works? *(Theoretical framework: perceived effectiveness)*

5. What is your overall impression of the program? *(Theoretical framework: affective attitude, intervention coherence)*

6. What has helped to make screening your patients for Chagas easier? What has worked well in the program? *(Theoretical framework: self-efficacy, perceived effectiveness)*
    a. Probe: What do you think has made the program successful? Both activities that you have done, that other people have done, and activities that have been done as part of the program.
    b. Probe: Educational activities for providers; community outreach to patients; reflexive confirmatory testing at the CDC, assistance from others within Strong Hearts?
    c. Probe: Would it help you to have an EMR prompt? Would you use an EMR order set targeted towards individuals born in Latin America?

7. What has been the most difficult part about screening your patients for Chagas? What has been challenging about the program? *(Theoretical framework: burden, opportunity cost)*
    a. Probe: What have been the challenges from the administrative side? What have been the challenges from the clinical side? (How have these obstacles changed and been addressed over time?)
        i. Laboratory? Testing? standing orders? EMR? Splitting/storing aliquot? Obtaining CDC confirmatory testing?
        ii. Administrative? Remembering to order the test? Deciding whom to test? Calling patients with results? Concern whether patient will be billed? How to bill? Prioritizing time to do testing vs patients' other clinical needs?
        iii. Referral to BMC? Scheduling follow-up visits/referrals to BMC for those who have tested positive? Ensuring that patients follow up at BMC? Logistics of helping patients get to BMC? Communication with BMC team members?
        iv. Clinician comfort with the disease; explaining disease to patient before ordering? Discomfort providing test results to patients? Answering questions about Chagas?
        v. Patient awareness of Chagas disease? Making sure family members (e.g. children) are tested?
    b. Probe: How much additional time do you feel like you have had to spend on tasks specific to Chagas screening?

8. Does the clinic routinely collect country of origin? *(Theoretical framework of acceptability: burden)*
    a. If so, who collects this information? (registration, provider, etc.)
    b. If not, what are the barriers to collecting this? (probe: perceived patient discomfort, lack of time, other), do you know why not?

9. What do you wish you had known at the start of this program that you know now? This could be about the program itself, the procedures to get testing done, the disease, or anything else related to Chagas disease or the Strong Hearts program. (*Theoretical framework: affective attitude*)

10. What suggestions do you have to improve the program? (*Theoretical framework: affective attitude, perceived effectiveness*)
    a. If another clinic were to roll this out, what would you want them to know (aspects that worked or did not work)?

11. Would additional support help you to screen more patients? *(Theoretical framework: burden, opportunity cost, self-efficacy)*
    a. Paper reminders? Additional personnel? Students?

12. What type of training or capacity development do you think would be useful for your department to increase knowledge, awareness and testing for Chagas disease, if any? *(Theoretical framework: perceived effectiveness)*

   a. Probe: grand rounds, webinars, SMS reminders about Chagas disease, circulating electronic versions of articles, social media? Role for CME? Information from health department?

Now I'd like to switch topics slightly and ask you a few questions about general professional educational activities.

13. What is the most important consideration when you are deciding whether or not to participate in an educational activity/training? *(Theoretical framework: opportunity cost)*

14. What would be the biggest motivator for you to attend a training on a topic you do not know much about? (examples: CME credit, patient population, personal experience, etc.) *(Theoretical framework: perceived effectiveness)*

15. What kind of educational activities have stuck in your mind well after they have concluded? What was it about them that makes you still remember them? (probe: in-person/zoom lectures - Grand Rounds, Department meetings, pre-conference workshops; Summary document/recorded material – Medscape; Emailed material – case series questions; other) *(Theoretical framework: affective attitude)*

16. What other educational resources do you make use of – both traditional and non-traditional? (probes: UpToDate, Facebook, Twitter, LinkedIn, Instagram, apps, webpages)

17. Is there any type of format that you really disliked and/or that was ineffective? If so, why? Did it make it more difficult to retain the information? *(Theoretical framework: affective attitude)*

18. How do you determine if information is credible or reliable? What makes you trust a source?

19. Do you prefer trainings that occur at a specific time (i.e. workshops, webinars, meetings) or do you prefer something you can access when convenient for you and go at your own pace (e.g., online trainings)? *(Theoretical framework: affective attitude)*

20. Do you have any questions, concerns or additional points you would like to raise?

21. Whom else should we contact to understand Strong Hearts better?

INSECT Focus Group Discussion Guide

Welcome, introduction and instructions to participants

Welcome and thank you for volunteering to take part in this focus group discussion. You have been asked to participate as your point of view is important. We realize you are busy, and we appreciate your time. My name is _______________ and assisting me today is ______________.

Introduction: The purpose of this focus group discussion is to hear about your views on novel and existing methods for learning and sharing clinical information, particularly as it relates to Chagas disease. Chagas disease is a parasitic infection, endemic to Latin America, and affects approximately 8-10 million people. Despite this, many healthcare professionals in the United States have limited knowledge about the Chagas. We are working on a project to increase awareness about Chagas among a range of healthcare providers in the US. We will be asking you some questions and also showing you some examples in order to better understand your preferences for how you like to learn clinical information. We will use the results of these focus group discussions to assemble a toolbox of engaging and practical resources that can be used and shared by healthcare providers from a range of disciplines. This focus group discussion will take no more than 90 minutes. May I record the discussion to allow for accurate transcribing?

Anonymity: Despite being recorded, I would like to assure you that the discussion will be anonymous. The recording will be kept safely in an encrypted computer folder and transcribed word for word. The transcribed notes of the focus group discussion will contain no information that would allow individual subjects to be linked to specific statements. Only the study team will have access to the recordings and the transcriptions. You should try to answer and comment as accurately and truthfully as possible. I and the other focus group participants would appreciate it if you would refrain from discussing the comments of other group members outside the focus group. If there are any questions or discussions that you do not wish to answer or participate in, you do not have to do so; however please try to answer and be as involved as possible.

Ground rules

The most important rule is that only one person speaks at a time. There may be a temptation to jump in when someone is talking but please wait until they have finished.

There are no right or wrong answers, these are all just ideas, experiences, and opinions. All of these are valuable to us.

You do not have to speak in any particular order.

When you do have something to say, please do so. There are many of you in the group and it is important that I obtain the views of each of you. Even just saying you agree with a previous statement is very helpful to us.

That being said, you do not have to agree with the views of other people in the group.

Does anyone have any questions?

OK, let’s begin.

Warm up

First, I’d like everyone to introduce themselves. Can you tell us where you work and what your role is there (physician, nurse, etc.)?

Introductory question

I am just going to give you a couple of minutes to think about any recent trainings or courses you may have attended, including but not limited to those where you may have received continuing education credit. Is anyone happy to give an example?

Guiding questions

1. What would be the biggest motivator for you to attend a training on a topic you do not know much about? (prompt if no replies: CME credit, patient population, personal experience, cost, etc.) *(Theoretical framework of acceptability: ethicality, perceived effectiveness, opportunity cost, burden)*

2. What kind of educational activities have stuck in your mind well after they have concluded? What was it about them that makes you still remember them? (probe: in-person/zoom lectures - Grand Rounds, Department meetings, pre-conference workshops; Summary document/recorded material – Medscape; Emailed material – case series questions; other) *(Theoretical framework: affective attitude)*
    a. Is there any type of format that you really dislike? If so, why? Did it make it more difficult to retain the information?

3. What other educational resources do you make use of – both traditional and non-traditional? (probes: Facebook, Twitter, LinkedIn, Instagram, apps, webpages) *(theoretical framework: affective attitude)*

4. How do you determine if information is credible or reliable? What makes you trust a source?

5. Do you prefer trainings that occur at a specific time (i.e. workshops, webinars, meetings) or do you prefer something you can access when convenient for you and go at your own pace? *(theoretical framework: opportunity cost, affective attitude)*

6. Now I’m going to show you a few examples of trainings or resources and I’d like to hear what you think about them. (show Rachel’s patient video, Twitter feed, case series email, GTEN EMR links. Ask about phone/computer preference for getting information) *(theoretical framework: affective attitude)*

7. Besides the types of materials I just shared or that we have already discussed, are there any others that you think would work well for sharing information with healthcare professionals? *(theoretical framework: affective attitude)*

8. What materials do you share with your patients to help them learn about a topic (videos, handouts, websites, etc.)? *(theoretical framework: affective attitude)*

9. Lastly, I'd like to present a screening program to you that is currently being used in Boston, Massachusetts. Briefly, the program runs as follows:

    a. If a provider in pediatrics, ob/gyn, adult internal medicine or family medicine has a patient from an endemic country, the provider explains the screening process and offers a screening test
    b. One sample is taken and divided into 2 aliquots: one is processed for screening, one is frozen
        i. If the screening test is positive then the frozen aliquot is sent to CDC for confirmation
            1. Probe after finished explaining process: Who might help to coordinate this? Need to unfreeze aliquot, package for shipping, etc.
            2. Test at CDC is free of charge
        ii. If the confirmatory test from CDC is positive, the patient is notified by a clinician in the clinic and referred to ID for evaluation and consideration of treatment

    Do you think this type of program could be rolled out in your health center or hospital? What do you anticipate might be barriers or challenges? *(Theoretical framework: affective attitude, intervention coherence, burden, opportunity cost, perceived effectiveness)*
            1. Probe: insurance issues? Follow-up/finding patients to give results? Referal network for ID? Resources for storing sample and then shipping?

10. What one factor would make you more likely to order a screening test? *(Theoretical framework: perceived efficacy, self-efficacy)*
    a. Probe: EMR prompt? Professional society's guidelines? Cost? Comfort/understanding of the disease and explaining testing and results?

Concluding question

Of all the things we've discussed today, what would you say are the most important things to keep in mind when developing new training materials?

Conclusion

Thank you for participating. This has been a very successful discussion. Your opinions will be a valuable asset to the study and we hope you have found the discussion interesting. If you have any questions or concerns, please feel free to contact me directly or the study at Chagas@bu.edu. Thank you again!
